# A review of current evidence on food allergies during pregnancy

**DOI:** 10.1002/fsn3.3451

**Published:** 2023-06-27

**Authors:** Tabussam Tufail, Yusra Rasheed, Huma Bader Ul Ain, Muhammad Umair Arshad, Muzzamal Hussain, Muhammad Nadeem Akhtar, Shamaail A. Saewan

**Affiliations:** ^1^ School of Food and Biological Engineering Jiangsu University Zhenjiang Jiangsu China; ^2^ University Institute of Diet and Nutritional Sciences The University of Lahore Lahore Pakistan; ^3^ Department of Food Science Government College University Faisalabad Faisalabad Pakistan; ^4^ Department of Food Sciences, College of Agriculture University of Basrah Basrah Iraq

**Keywords:** allergens, allergy, food allergy, foods, pregnancy

## Abstract

Food allergy is the reaction of the immune system of the body that occurs after consuming specific foods. During specific physiological ages of pregnancy, women are more prone to different allergic reactions and mostly these reactions may prolong and have long‐term effects. The hypersensitivity of different types of allergens is mainly linked with the adversity of reactions. The chances of suffering food allergies in women are greater than in men; women are usually more prone to get allergic to some foods during their specific physiological age of pregnancy. Food allergies are more common in pregnant women as every fifth pregnant woman is affected by some kind of allergy. The specific reasons and evidence of the causes of these food allergies during pregnancies have yet to be explored. A pregnant woman should take a balanced diet and avoid consuming known allergic foods to minimize the risk and complications. This review aimed to broaden the knowledge on food allergies during pregnancies, their onset in the babies, and to make it easy for pregnant women to cope with the complications caused by these food allergies. It also aimed to figure out the certain food that might be responsible for the onset of allergies in women during pregnancy and the effect of these allergies on their babies.

## Graphical abstract

Food allergy is the reaction of the immune system of the body that occurs after consuming specific foods. Food allergy and food intolerance are commonly confused since food intolerance symptoms often resemble those of food allergy. Allergies are frequent during pregnancy, but not all pregnant women who have them are long‐term suffers. The extent of responses to the allergies depends upon the hypersensitivity to the allergen. This review aimed to broaden the knowledge on food allergies during pregnancies, their onset in the babies and to make it easy for pregnant women to cope with the complications caused by these food allergies. And to figure out the certain food that might be responsible for the onset of allergies in women during pregnancy and the effect of these allergies on their babies.

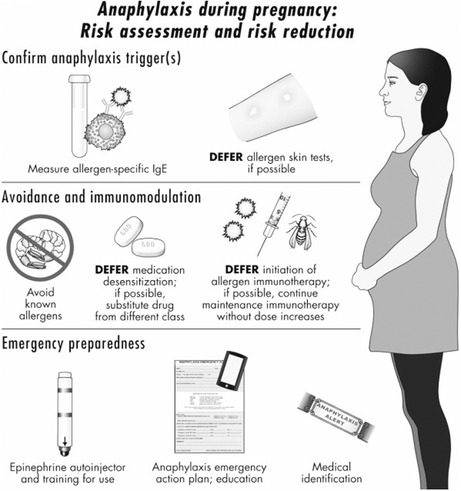

## 1 INTRODUCTION

Allergens specifically food allergens are considered to be a major health concern now a day. Epidemiological studies showed that during the past two decades, our food consumption pattern resulted in an increased prevalence of different types of food allergies which also resulted in higher persistence and severity of clinical symptoms (Di Costanzo et al., 2022). An adverse food reaction is any irregular reaction that occurs after consuming the food. Two types of adverse food reactions are food hypersensitivity which includes food intolerance and food allergy, and food aversion which is a psychological avoidance of an unfavorable reaction conditioned by Pavlovian conditioning (Tuck et al., 2019). Problems in food allergies are starting from eczema to life‐threatening allergies. Common types of allergic reactions may arise by consuming foods from shellfish, peanuts, fish, tree nuts, wheat, cow's milk, soy, and egg origin (Altman et al., 2020). At special physiological age of pregnancy, pregnant women are more prone to different allergic reactions. These symptoms are only reported by women who have never had allergies before (Murkoff, 2016). During the pregnancy, the maternal immune system develops an interface between Th2 and Treg environment rich in cytokines such as IL4, IL10, IL13, and TGF‐β to protect the fetus from rejection against antigens (Pastor‐Vargas et al., 2016). The food the mother eats during pregnancy might contribute to her and also the child's immunological profile (Devereux, 2006). Food allergens present in mother's diet, which are considered to be very stable proteins, affect the fetus by crossing the placenta, resulting in constituting the first route of sensitization. The present study aimed to figure out certain foods that cause allergies to the mother during pregnancy and to find substitutions for those foods that trigger allergies to the mother during pregnancy.

## 2 ALLERGY

Allergy is a Greek word, *allos*, which means “other word,” and *ergon* means “action,” which means a state of the organism, or the body, characterized by changes in response. Nowadays, allergic conditions are increasing day by day in the world. Conditions are not limited to one organ. They may affect different organs and, in this way, affect several organs at the same time (Skypala, 2019).

### 2.1 Types of allergies

Allergy is the result of numerous genetic and environmental factors, resulting in a lack or loss of tolerance to certain agents. Infants and children are at greater risk than adults for developing different types of allergies such as drug allergy, food allergy, skin allergy, dust allergy, allergy to insects, latex allergy, allergy to molds, pet allergy, and pollen allergy. Allergies are categorized according to their cause, severity, and management and avoidance options. An anaphylactic type or immediate reaction is included in type I hypersensitivity, which included allergies to pollen, foods, drugs, stings of insects, etc. Specific antibodies are involved in type II hypersensitivity as shown in Table 1. The antibody binds to and destroys the cell to which it is attached. When the body refuses to recognize the donated organ as its own following an organ transplant, this reaction occurs. Type III hypersensitivity is a reaction mediated by the immune complex. An immunological complex is a combination of an antibody and an antigen that has been bonded together. This ends up in a cascade of reactions within the body that works to destroy local tissues. Glomerulonephritis and systemic lupus erythematosus (lupus, SLE) are examples of this problem. Type IV hypersensitivity is a delayed or cell‐mediated reaction caused by T‐cell lymphocytes, which are unique immune cells. The T cells are taken for mounting an allergic response from a few hours to a few days. Examples include contact dermatitis like poison ivy rashes (Loh & Tang, 2018). Types of hypersensitivity reactions and their characteristics are also presented in Table 1.

**TABLE 1:** Types of hypersensitivity reactions and their characteristics.

Type of hypersensitivity	Medicated by	Mechanism
I	IgE	Mast cells and basophil
II	IgG, IgM	Phagocytes, complement and interference with cell function
III	IgG	Immune complexes, phagocyte, and NK cell activation
IV	T cells (TH1, TH2, and cytotoxic T cells)	Macrophage and eosinophil activation and cytotoxicity

### 2.2 Prevalence of allergy

The global burden of allergy varies depending on the types of allergies, for example, the prevalence of asthma varies from 1% to 20%, allergic rhinitis varies from 1% to 18%, atopic dermatitis varies from 2% to 10%, and food allergy varies from 1% to 2%. On the other hand, self‐reported prevalence of food allergy is up to 37%. The literature shows a vast variation among adults while a much higher prevalence of allergic reactions was observed in children (Dierick et al., 2020). In a study conducted by Luo and coworkers, it was observed that the prevalence of allergic diseases is about 10% in infants whose first‐degree relatives do not have allergic diseases compared to 20% to 30% in those having allergic reactions among first‐degree relatives (Luo et al., 2022). These allergic reactions may affect the quality of life which also affect the socio‐economic condition of a family. All these contributing factors negatively affect the work productivity, mostly due to presenteeism.

## 3 FOOD ALLERGY

Food is any substance whether raw, semi‐processed or processed that is projected for human consumption. It includes drinks, food additives, chewing gums, or dietary supplements (Han et al., 2012). A food allergy is caused by the immune system when it targets harmless food proteins. The immune system is composed of different cells as well as proteins and it is the part of the body that normally fights against germs. Food allergy is a multifaceted disorder of immune functions and is caused by specific genetic variations combined with certain environmental and nutritional factors. Genome‐wide association studies have found certain loci for food allergy, including genes involving barrier integrity (filaggrin and serine protease inhibitor), immune function, and others. However, the increase in food allergy is too rapid to be due to genetics alone, and migration studies show us that these increases can occur in a single generation. The worldwide prevalence of food allergy is increasing day by day, which ultimately emphasizes the need for better policies for prevention, diagnosis, and treatments. During the past few decades, scientists have made pronounced efforts in understanding the causes and mechanisms resulting the food allergies. Previously, efforts were made to avoid common food allergens during the special physiological age of pregnancy and lactation and also delay the utilization of allergic foods in children. Current evidence highlights the use of diverse food groups without eliminating or increasing the consumption of allergic foods during pregnancy or lactation to avoid or reduce the chances of allergies arising from food (Sampath et al., 2021). The immune system “attacks” proteins in meals in two ways: by releasing chemicals from cells or by creating IgE (immunoglobulin E) antibodies, which are proteins. When allergic reactions occur immediately after a food is consumed, the most common types of food allergy are the product of the immune system producing the IgE antibodies. These proteins are like small antennas sitting on allergy cells (called mast cells and basophils) and detecting the proteins in the food. The “antennae” IgE will detect, for example, a peanut protein or an egg protein, a particular food protein (Sicherer, 2017).

### 3.1 Food allergens

Food allergens are some specific ingredients or components of foods that are present within the food (normally proteins but at times they may be chemical haptens) that are detected by allergen‐specific immune cells and cause‐specific immunological reactions that cause specific symptoms (Han et al., 2012). Some raw foods, such as apples, induce allergies, while others are rarely consumed without some form of food processing. Peanut is an example of the latter type and they are commonly consumed after being roasted. Other allergenic foods are pasteurized or sterilized to extend their shelf life. Sterilization and pasteurization of dairy products and milk are good examples. The food business today employs a wide range of food processing technologies, including heating, cooling, high‐pressure treatment, ultra‐filtration, irradiation, hydrolysis, and fermentation (van Hengel, 2007).

#### 3.1.1 Prenatal exposure to food allergens

The key questions now are around dietary allergy exposure in both maternal and newborn diets. Until recently, the American Academy of Pediatrics (AAP) recommended that families with an infant at increased risk of atopy based on family history avoid peanuts in the infant's diet for the first 3 years of life and wait until the first (milk), second (egg), or third (tree nuts and fish) years of life to introduce common food allergens. It was also suggested that during pregnancy and breastfeeding, mothers should avoid peanuts and additional allergens. In the United Kingdom, similar suggestions are still in place regarding peanut avoidance (Kent et al., 2015). There is very little evidence‐based guidance on when to introduce this allergen into the diet and whether to introduce it regularly or irregularly these foods in small or large quantities. This reflects a significant gap in our knowledge of weaning infants. To prevent allergy at the global‐level World Health Organization plan to promote the first 6 months of the infant's life exclusive breastfeeding and thus delay weaning onto milk formulas and solids (World Health Organization, 2014). The conventional knowledge is that during pregnancy and lactation, early exposure to allergenic foods could lead to food allergies. Prevention strategies during early childhood, breastfeeding, and pregnancy are to eliminate allergenic food proteins from the diet (Lack, 2008).

### 3.2 Prevalence

Food allergy is a major public health issue that affects both adults and children around the world and is recognized as a serious global health concern (Han et al., 2012). The prevalence of allergic reactions to food varies greatly in developed countries where people can afford a variety of food; 1 in 10 people may be affected by food allergies while the rates are increasing in developing countries (de Silva et al., 2020). Currently, food allergy is affecting 5%–8% of world's population and the rate of allergic reactions to food is increasing as rapidly as 1% in a decade (Fujimura et al., 2019). It is important to note that some individuals strictly avoid potential allergen foods, which ultimately resulted in impaired quality of life. All these incur extra financial load associated with management of chronic food allergic reactions (Warren et al., 2020).

### 3.3 Common food allergies

Allergic reactions have been categorized into four major types depending upon pathogenesis mechanisms. Type I reactions are immune‐mediated adverse reactions to foods and are categorized by the development of IgE against food allergens. Symptoms can include the inflammation reactions normally initiated by cellular components and mediated by eosinophils and T cells. IgE‐associated food allergy is diagnosed through serum and other body fluids for food allergen‐specific IgE, and through in vivo cellular IgE‐mediated processes (Valenta et al., 2015).

**FIGURE 1:**
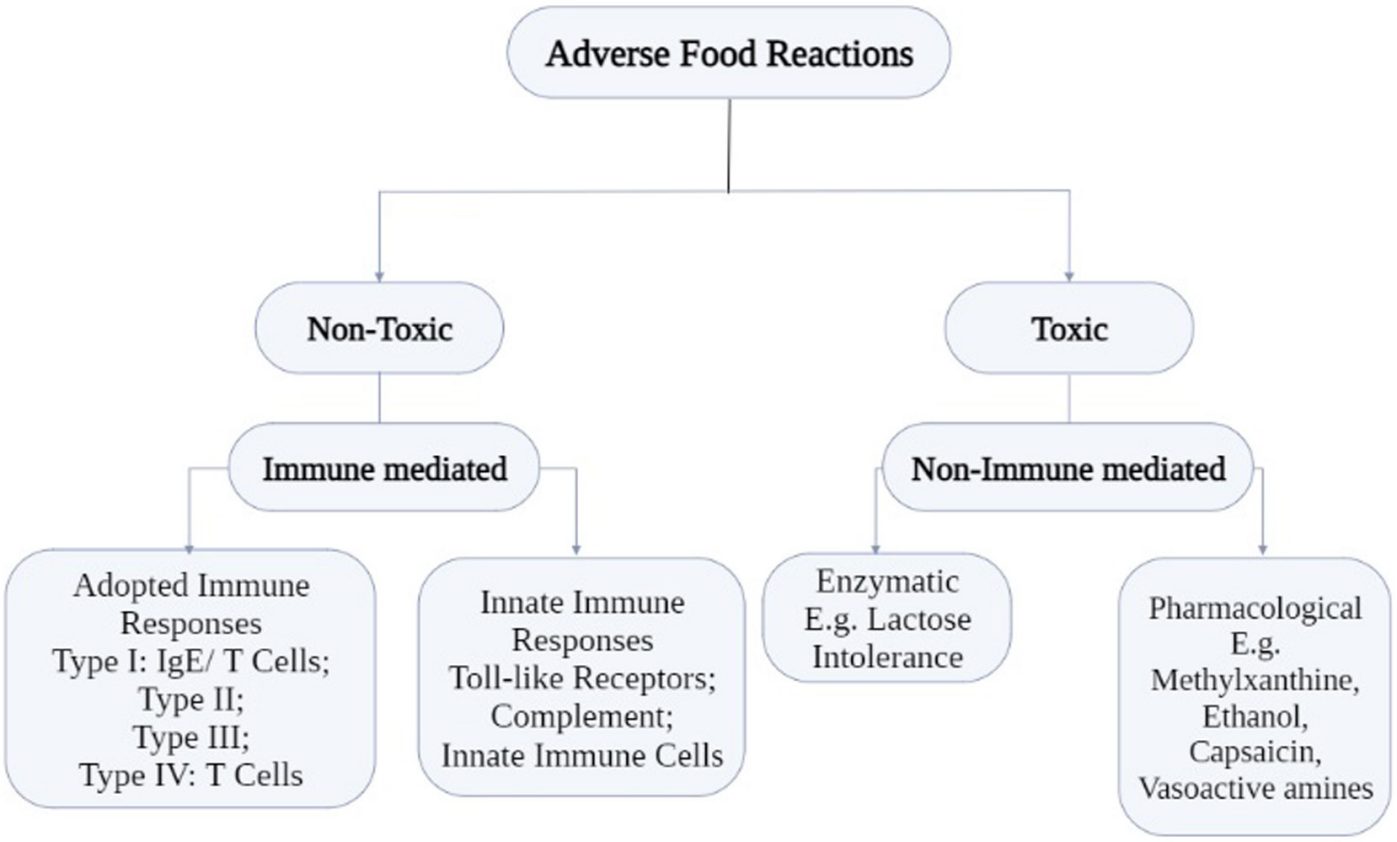
Types of adverse food reactions.

Although it is tempting to speculate that food antigen‐specific IgG can cause adverse reactions via type II or type III hypersensitivity, there is no solid experimental evidence to support the relevance of these reactions to food allergies that develop in patients (Figure 1). Accordingly, several position papers strongly recommend against testing for food antigen‐specific IgG in the diagnosis of food allergy.

The role of environmental factors toward the susceptibility and incidence of food allergic reactions are understood by epigenetic mechanisms (Potaczek et al., 2021). Environmental factors in combination with nutritional factors influence a vital part during pregnancy and the first thousand days of life in determining susceptibility of an individual toward the development of chronic non‐communicable diseases later in life, such as allergies (Acevedo et al., 2021). Therefore, during the gestational period and infancy, immunity of the individual is mainly contributed by nutritional factors through epigenetic mechanisms (Paparo et al., 2014). So, if the individual is affected by certain sorts of food allergies, he/she might be at higher risk of developing other types of allergies later in life, and other conditions mainly as a result of a dysregulation of the immune system (Di Costanzo et al., 2022; Yang et al., 2020).

### 3.4 Symptoms

Food allergy's initial symptoms are often sneezing, runny nose, itching, burning and swelling around the mouth, dermatitis, rashes, and difficulty in breathing. Slightly severe allergic reactions include eczema, hives, and hay fever. In severe cases, allergic responses may cause gastrointestinal and respiratory distress that may require immediate emergency intervention. Severe allergies to environmental or dietary allergens, as well as medication, can cause life‐threatening hypersensitivity reactions that can be deadly in some people. Getting sudden allergic symptoms within 1 or 2 h of consuming a portion of food may increase suspicion of an allergy to the food. However, allergic reactions can also be more insidious. A food allergy may cause severe daily symptoms that can be harder to relate to particular foods. Women may feel uterine contractions. When blood circulation is disturbed, the heart may beat very rapidly or very slowly, the skin may become pale or blue, the pulse may be difficult to feel, blood pressure may be weak, and discomfort, dizziness, lightheadedness, and passing out may happen. An individual often gets a feeling of inevitable doom in some kind of serious allergic reaction (Renz et al., 2018).

**FIGURE 2:**
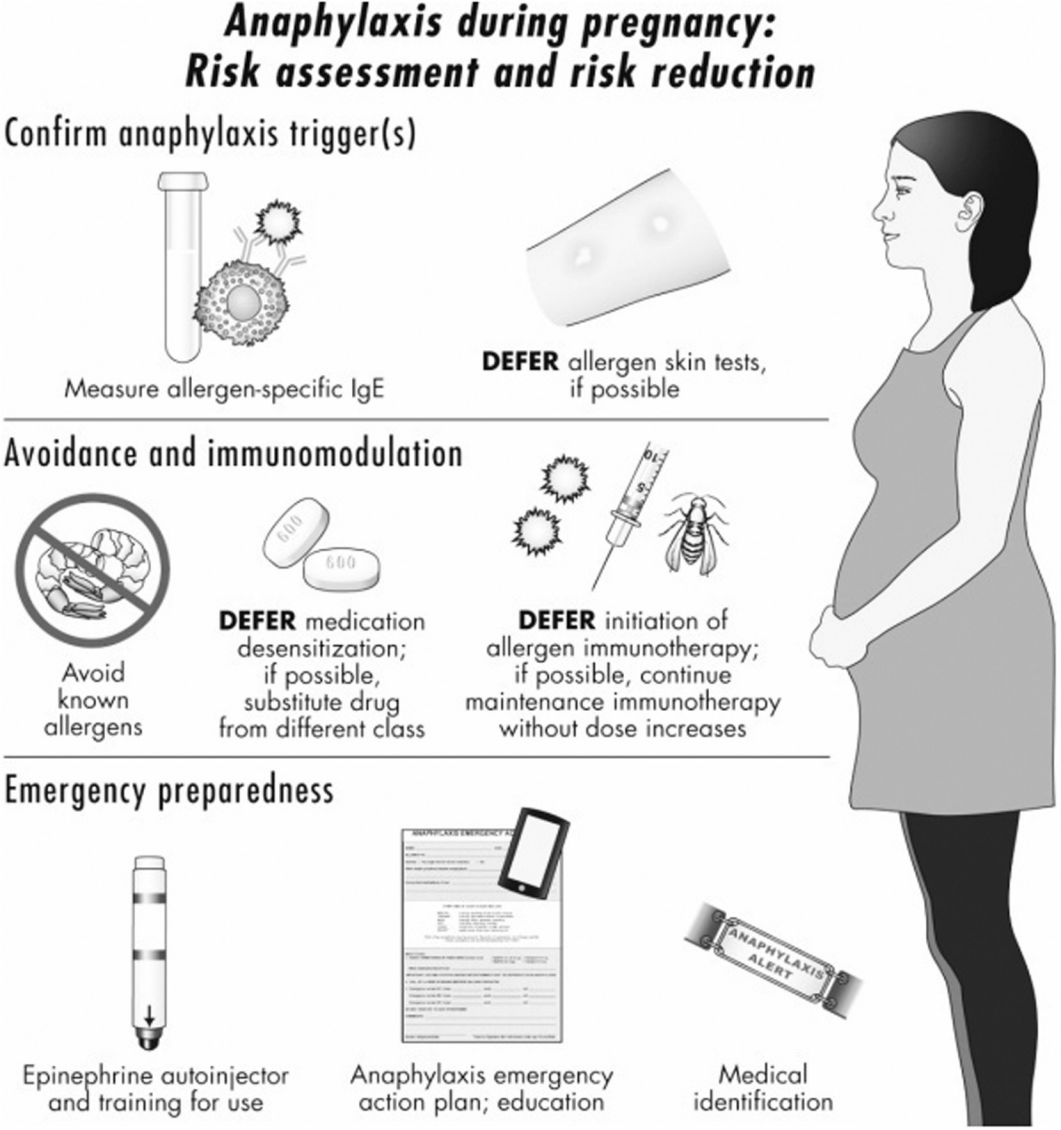
Different types of symptoms that occur after anaphylactic reaction during pregnancy. Source (Simons, Schatz, & immunology, 2012).

An allergic reaction known as anaphylaxis is a life‐threatening condition. It has a quick onset and might be fatal (Clark & Camargo Jr, 2007). Case ascertainment is critical in pharmacovigilance since it can occur after the administration of medicines and vaccines. Anaphylaxis is defined by the involvement of the respiratory or cardiovascular systems as part of a multisystemic hypersensitivity reaction, with significant broncho spasm as a key characteristic (Erlewyn‐Lajeunesse et al., 2010). Egg and cow's milk were the most common foods causing anaphylaxis in infants (Samady et al., 2018). After exposure, a trigger symptom of an anaphylactic reaction normally occurs within seconds to minutes. However, after exposure, some symptoms occur after 2 h. Different types of symptoms that occur after an anaphylactic reaction are presented in Figure 2. Skin symptoms include flushing, itching, swelling, and hives. Symptoms in gastrointestinal tract include cramping in the abdomen, vomiting, abdomen pain, nausea, and diarrhea. Respiratory symptoms include wheezing, congestion in the nose, tightness in the chest, runny nose, shortness of breath, and cough. Low blood pressure, dizziness, fast heart rate (tachycardia), and lightheadedness are cardiovascular symptoms of anaphylaxis (Kasper et al., 2015).

### 3.5 Diagnosis

There are several techniques for diagnosing allergic reactions to food and other substances. Two common diagnostic tests for determining the allergens are the “Skin prick test” and the “Blood test.” The skin prick test is the most popular test for allergy diagnosis (SPT). The most common test for allergy diagnosis is the skin prick test (SPT). SPT is frequently the first‐choice test in the diagnostic workup for allergic illnesses because of its reliability, safety, convenience, and low cost. It is conducted by pricking the skin with a lancet through a drop of an allergen extract, usually on the volar surface of the forearm. SPT is a non‐invasive test that may screen numerous allergens in 15–20 min (Frati et al., 2018). Another method for evaluating food allergies is a blood test. A RAST test (radioallergosorbent test) is used to determine what substance a person is allergic to. It detects the presence of IgE antibodies to a particular allergen. Hundreds of allergens may be checked from a single blood sample, and the test covers food and inhalant allergies. The basophil activation test (BAT) is a valuable technique for establishing a diagnosis in a variety of allergy instances where traditional diagnostic instruments have failed to correctly identify the allergen. For example, this test can help determine a diagnosis in individuals with a history of systemic sting reactions, negative skin, and sIgE tests, and a hint of potential mastocytosis. As a result, it can only be used as a screening test for VIT in a few cases of sting‐induced anaphylaxis (Pitsios et al., 2022).

### 3.6 Treatment

Now strict and careful avoidance of allergic food is recommended for the management of food allergies (Sampson et al., 2014). Avoiding swallowing the causal allergens is the mainstay of treatment for IgE‐mediated food allergies. If an allergen is mistakenly consumed, epinephrine, antihistamines, and steroids may be used to treat allergic responses. The use of cross‐reactive allergens or allergen‐specific immunotherapy is not recommended by NIAID. On the other hand, the idea of promoting tolerance in children with food protein allergies through regulated exposure to allergens (desensitization), which was first advocated 70 years ago, has lately been resurrected (Guandalini & Newland, 2011). Furthermore, evidence suggests that including baked milk in the diet of children who tolerate such foods speeds up the development of unheated milk tolerance as compared to strict avoidance (Kim et al., 2011). Oral desensitization treatment for food allergies in children appears to be a promising breakthrough at the moment, but additional research is needed before this approach can be standardized and appropriate recommendations given (Land et al., 2011).

#### 3.6.1 Oral immunotherapy

OIT has been studied as a prospective therapy option for food allergies in the past and more recently for more than a decade. Studies have been conducted on multiple food allergens but most randomized controlled trials have focused on eggs, peanuts, and milk (Anagnostou et al., 2014). In this immunotherapy, an allergen powder (e.g., contains peanut protein along with carbohydrates and lipids) ingestion is recommended daily, which is mixed with other food and ingested. OIT is administering progressive dosages of the offending food to patients in the hopes of gradually developing desensitization or, in the worst‐case scenario, SU. Immunotherapy is thought to work by modulating the immune response, which includes a shift from allergen‐specific IgE to IgG4 and a decrease in basophil activation to allergen cross‐linking, as well as an increase in regulatory T cells (Kulis et al., 2011). OIT has been linked to the best clinical and immunological outcomes of any therapy strategy, including desensitization and, in some cases, SU, as well as considerable immunological regulation (Burks et al., 2018).

### 3.7 Environmental risk factors for food allergy

Environmental factors greatly influenced the development of allergies.

#### 3.7.1 Prenatal factors

The infant can be born highly sensitized to food allergens because they cross the human placenta in concentration and it can stimulate the fetal lymphocytes. Only a small number of studies have evaluated that the effect of maternal diet during pregnancy can develop an allergy in newborns. A study was conducted in which 212 pregnant women enrolled; 28 weeks before delivery, milk and egg were prohibited from the diet of 104 women, and the remaining 108 had a normal diet. Physical examination, parental questionnaires, skin prick test, and total IgE levels were evaluated in the offspring. By the age of 18 months, no statistically major difference was noted in infants and there was no occurrence of milk or egg allergy in both groups. In a follow‐up of these children, no difference was noted at the age of 3 and 5 years (Kaza et al., 2007).

### 3.8 Genetic and molecular risk factors

Individual food allergies, such as peanut allergies, have been on the rise in some regions of the world during the last decade. The prevalence of new food allergies, especially sesame seed allergy and kiwi allergy, is increasing. Although genetic risk factors are unlikely to account for the recent increase in food allergies, there are likely to be genetic predisposing factors for the development of food allergies, just as there are genetic predisposing factors for the development of other atopic illnesses (asthma and eczema). It has to be investigated whether the same genetic polymorphisms related to asthma and eczema are present in food allergy patients, or whether there are unique polymorphisms connected with food allergies (Lack, 2008).

## 4 FOOD ALLERGY DURING PREGNANCY

**FIGURE 3:**
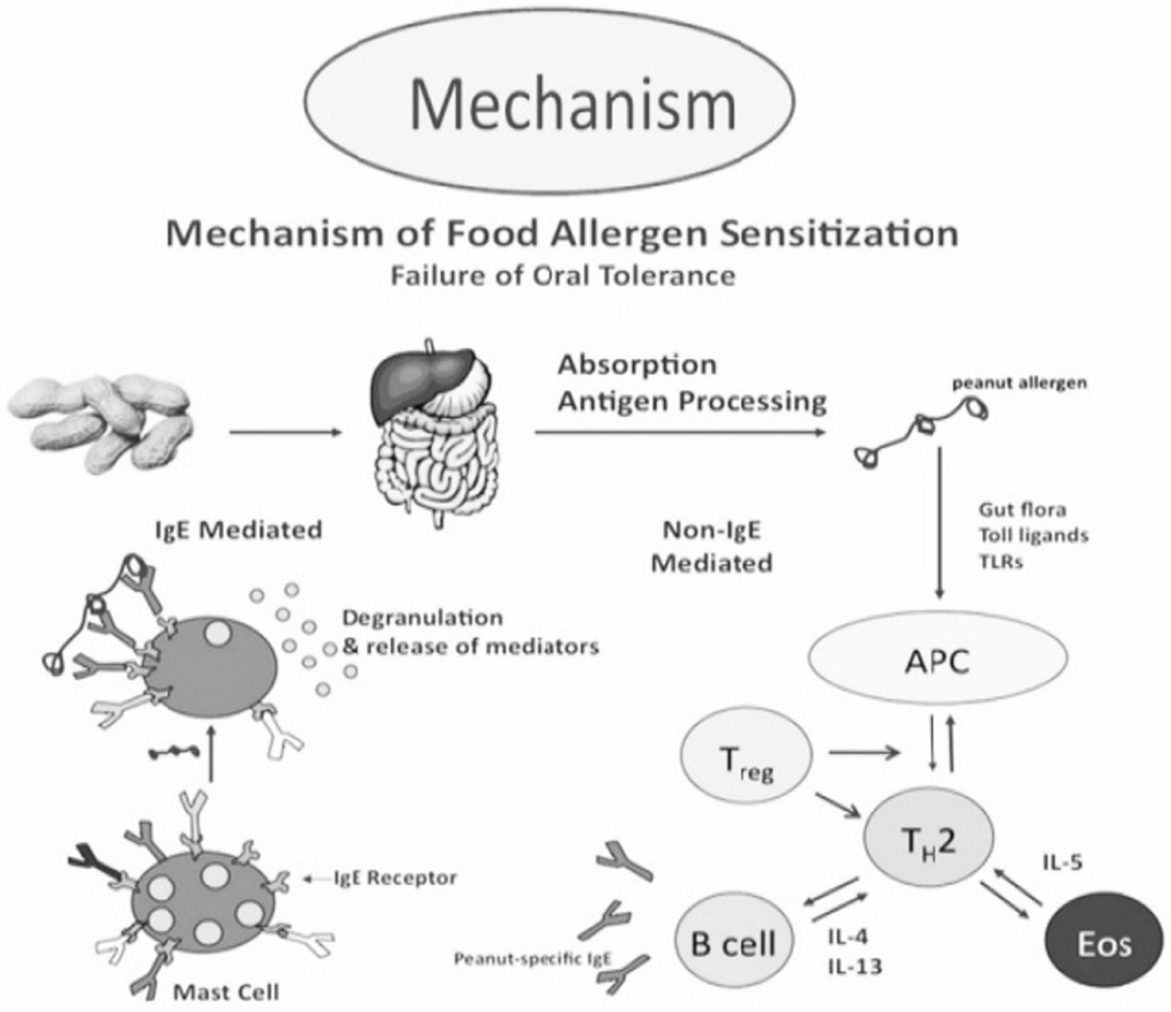
Mechanism of food allergen sensitization.

Allergic symptoms that occur before pregnancy may either be diminished or encouraged during pregnancy with equal frequency. To ensure the welfare of the baby and mother, diagnosis and management of food allergy, asthma, and optimal allergy are essential during pregnancy (Pali‐Schöll et al., 2017). In industrialized countries, allergic sensitization to common allergens can be identified in about 25% to 35% of the general population (Braun‐Fahrländer et al., 2004). The mechanism of food allergen sensitization is shown in Figure 3.

### 4.1 Prevalence of food allergy during pregnancy

Allergic diseases, especially rhinitis in the United States, are about 18% to 30% in women of childbearing age (Incaudo, 2004). Drug allergy, conjunctivitis, food allergy, acute urticaria, and anaphylaxis are some allergic diseases that can complicate pregnancy. Such disorders constitute the most common section of health problems that make pregnancy more complicated. The course and outcome of pregnancy can affect by asthma and allergic disorders. Pregnancy itself may also affect the course of asthma and other diseases (Schatz et al., 2003).

### 4.2 Diagnosis of allergy during pregnancy

Allergy diagnosis in pregnant women may rely on a comprehensive medical history and review of the symptoms. For diagnosis, it may be helpful to have a log of allergy symptoms and (ii) avoidance of known allergens followed by recording changes in allergic symptoms. It must be emphasized that it is necessary not to put the mother on a strict diet restriction for diagnosis of food allergy, as this could have a negative effect on the nutritional status of both the mother and the developing infant (Pali‐Schöll et al., 2017). In vitro diagnostic methods such as serological tests for IgE common to allergens, for example, Immuno‐CAP or radioallergosorbent test (RAST) or lymphocyte transformation test for type IV allergy diagnosis, are preferred to skin and provocation tests that should be delayed until after birth due to possible, but uncommon, anaphylactic reactions (Simons & Schatz, 2012). The same applies to food tests and other research on difficulties. Although there are no documented adverse effects of patch testing during pregnancy or breastfeeding, most physicians advise against use as a general precaution because test findings can interfere with immunological changes caused by pregnancy (Lazzarini et al., 2013).

### 4.3 Management of Allergic Diseases during pregnancy

Allergic mothers should prevent exposure, intake, and interaction with known allergens that have been identified. In particular, patients should also avoid inhaling any potent asthma causes, such as irritating pollutants, animal dander, smoke, house dust, and tobacco (Oykhman et al., 2015). New findings suggest that immunotherapy with allergens not only improves the disease in the pregnant patient but that this procedure may also avoid allergic sensitivity in the infant. Nonetheless, further studies are required to confirm the impact of immunotherapy with allergies during pregnancy on the child's sensitization growth (Lieberman, 2014).

#### 4.3.1 Maternal diet during pregnancy

During pregnancy, the food that is consumed by the mother can contribute to the immunological profile of the child and herself (Devereux, 2006). It was studied that the Japanese diets that mainly consisted of seaweed, vegetables, fruits, minerals, fiber, and antioxidants were mainly associated with a lower risk of different allergic reactions like lowering the risk of allergic rhinitis in pregnant women (Miyake et al., 2006). Intake of different minerals like magnesium, calcium, and phosphorus at the special physiological age of pregnancy may also be associated with decreased allergic reactions. Excessive use of vitamin D may result in different allergic reactions, while on the other hand deficiency of vitamin D due to low exposure to sunlight indirectly resulted in asthma‐like allergic reactions in pregnant women (Wjst & Hyppönen, 2007; Camargo Jr et al., 2007). National Institute of Allergy and Infectious Diseases (NIAID) Guideline #36—The maternal diet should not be restricted during pregnancy or breastfeeding as a strategy for preventing the development or clinical course of food allergy. Fellow of the American Academy of Allergy Asthma and Immunology advised that during pregnancy women should consume regular diets keeping in mind the additional nutritional needs required for proper development and growth of the fetus. Some work has been done to look at intakes of specific nutrients during pregnancy with reduced risk of allergies in offspring. A lot of attention has been paid to vitamin D, omega‐3 polyunsaturated fatty acids (PUFAs), and folate (or the synthetic form, folic acid) (American Academy of Pediatrics Committee on Nutrition, 2000).

#### 4.3.2 Peanut restrictions of food during pregnancy

Allergies to peanuts are very general and can cause serious and potentially fatal allergic reactions. The two conditions, however, are considered distinct, since peanut is a legume. Many with peanut allergies, however, are also allergic to tree nuts. Although the reason people develop an allergy to peanuts is not clear, people with a family history of peanut allergies are considered to be most at risk. Because of this, it was previously thought that the introduction of peanuts through the diet of a mother breastfeeding or during weaning could induce a peanut allergy. On the other hand, many studies have shown that introducing peanuts early may be protective (Anvari et al., 2017). To prevent the development of allergy disorders in newborns with atopy in first‐degree relatives (high‐risk infants), the international guidelines suggested avoiding allergenic foods during breastfeeding and pregnancy. Furthermore, it was suggested that allergic supplementary meals be avoided until the age of 12 months, eggs until the age of 2 years, and peanuts, tree nuts, and fish until the age of 3 years. There is no solid evidence that a maternal exclusion diet during pregnancy has a protective effect (Høst et al., 1999). According to the Department of Health, peanuts should be avoided by atopic pregnant women, breastfeeding moms, and their children. The Department of Health, on the other hand, modified its recommendation in 2009. They discovered no indication that consuming or not eating nuts while pregnant affected the likelihood of a child having an allergy. Since then, they have stated that pregnant or breastfeeding women do not need to avoid nuts. There are no limits on the diet for mothers during pregnancy and lactation, according to the latest food allergy and anaphylaxis recommendations of the European Academy of Allergy and Clinical Immunology. Exclusive breastfeeding is recommended for the first 4–6 months of life, thereby preventing allergies from developing. For high‐risk children, hypoallergenic formulas with demonstrated preventative effects are indicated if nursing is not available (Valenta et al., 2015). The current data recommend that if women like eating peanuts or peanut‐containing foods during pregnancy or breastfeeding, then they may choose to eat peanuts as part of a safe balanced diet unless they are allergic to peanuts.

#### 4.3.3 Nutrient intake during pregnancy with food allergies

Unlike common opinion, pregnant women do not have to “eat for two.” The body gets more efficient at utilizing the energy and nutrients obtained from food, thus eating healthily is essential. However, later in pregnancy, the body has higher calorie requirements as well as increased needs for certain essential nutrients. RNI stands for ‘Reference Nutrient Intake’. RNI estimates are population dependent and not individual needs. RNIs should not be confused with RDAs (“Recommended Dietary Allowances”) although sometimes identical. The energy intake of non‐pregnant women is around 2000 kcal, while in pregnancy extra 200 kcal is required in the third trimester only. The folic acid requirement for non‐pregnant women is 200 mcg, while in pregnancy, an extra 400 mcg is a must in the first trimester and an extra 100 in the second and third trimesters. Proteins for non‐pregnant women are 51 g while for pregnant women additional 6 g are required. The need for vitamins also increases during pregnancy. Non‐pregnant women require 40 mg of vitamin C while for pregnant women, an extra 10 mg is required in the third trimester only. For non‐pregnant women, vitamin D requirement is 0 if they gained it from the action of sunlight on the skin. In pregnancy, 10 mcg is required. 600 mcg of vitamin A is the requirement of non‐pregnant women, while pregnant women need an extra 100 mcg. This shows a comparison between the nutrient requirements of a pregnant and a non‐pregnant woman (Webster‐Gandy et al., 2020). If a woman is suffering from any food allergy, it is of great concern to consume an adequate diet during pregnancy for a healthy baby. During pregnancy, allergic reactions and symptoms can fluctuate due to hormonal changes. Some women find their symptoms reduced by pregnancy; others find they increase.

##### Milk/dairy allergy

If a woman is suffering from lactose intolerance or cow's milk allergy, it is important to use other calcium‐containing alternatives instead of milk/dairy products. Calcium requirements in pregnancy do not increase, but two to three portions of dairy alternatives are important each day to meet requirements. Lactose‐intolerant women should include the non‐dairy alternative of milk in their diet, such as almond milk, soy, and rice to compensate for some of the losses due to not consuming whole milk. Before consuming it is best to check the label to make sure that they have added calcium (McCance & Widdowson, 2014). If a pregnant woman is suffering from milk allergy, consuming an adequate amount of calcium is essential for building the baby's developing bones and keeping the pregnant woman strong throughout pregnancy and during the period of lactation by keeping herself as well as the infant nutritionally healthy. The pattern of diet consumed by mothers during pregnancy and lactation influence and modify the risk of allergy in the offspring (Stråvik et al., 2020).

##### Egg allergy

Two to three portions of protein‐rich food are important to include in the diet every day during pregnancy. The egg is considered a major source of iron and protein. If suffering from an egg allergy, it is instructed to include alternative sources of protein like chickpeas, meat, lentils, fish, beans, and poultry (Coletta et al., 2010).

##### Fish/seafood allergy

The benefit of having oily fish once a week in a diet is well reported because of the omega‐3 fatty acid content. Foods containing omega‐3 fatty acids are important to eat because they help to support the development of a baby's brain and growth. Plant‐based sources of omega‐3 fatty acids are recommended to be included in the diet, for example, green leafy vegetables, linseeds, avocados, nuts, and seeds (McCance & Widdowson, 2014).

Allergens present in food products can be detected by different types of kits as discussed in the Table 2.

**TABLE. 2:** Allergens present in food products, symptoms of food allergy, and detection of these allergens.

Allergy	Allergens	Antibodies	Symptoms	Testing	References
Egg allergy	Ovomucoid; Ovo transferrin; Conalbumin; Lysozymes; Ovalbumin; Alpha‐livetin; Vitellus	IgE	Respiratory or cardiovascular symptoms, presenting with cough, wheezing, breathing difficulty, or pallor and floppiness are uncommon	Skin prick test, egg‐specific IgE, and egg component testing	‐
Fish allergy	Parvalbumins; Gelatin; Enolase; Aldolase; Vitellogenin; Tropomyosin	IgE	Anaphylaxis, nausea, gastrointestinal, and rhinitis	Skin prick test, Immuno‐CAP, and immunoblotting	‐
Peanut allergy	Ara h 1 and Ara h 3 (members of the cupin superfamily) and Ara h 2 and Ara h 6 (members of the prolamin superfamily)	IgE	Skin (e.g., urticaria, flushing, and angioedema), gastrointestinal tract (e.g., vomiting, nausea, abdominal pain, and diarrhea), respiratory system (e.g., rhinorrhea, sneezing, coughing), and the cardiovascular system (e.g., cardiovascular collapse)	Skin prick test and basophil activation test	‐
Cashew nut allergy	Vicilin; Legumins	IgE	Respiratory and gastrointestinal indications	Skin prick test, double‐blind placebo‐controlled food challenge (DBPCFC)	‐
Milk allergy	Casein protein and whey protein; alpha‐lactalbumin and beta‐lactalbumin	IgE	Hives; wheezing; Itching; or tingling feeling around the lips or mouth; swelling of the lips, tongue, or throat; coughing or shortness of breath; vomiting; and other digestive‐related problems	Skin‐prick test; blood test; oral food challenge	Ramachandran et al., 2020; Fiocchi et al., 2022

## 5 ORAL ALLERGY SYNDROME (OAS)

It is important to consider the intake of vitamin C which may be low because avoiding some vegetables and fruits due to food allergy during pregnancy. In pregnancy, the need for vitamin C increases during the last trimester. People having OAS can eat well‐cooked vegetables and fruits because cooking destroys the allergens. On the other hand, cooking also decreases the amount of vitamin C in the food. Pineapple, citrus fruits, banana, and other tropical fruits are rich sources of vitamin C. If the fruit and vegetable intake is low, or cooked fruits cannot be tolerated, it is instructed to consider taking a supplement containing vitamin C. Single vitamin supplements contain high doses, so it is best to avoid them as they can be dangerous in pregnancy (Coletta et al., 2010).

## 6 FOOD ALLERGIES IN PREGNANT AND NON‐PREGNANT WOMEN

Food allergies are on the increase. According to a study, the highest rates of intolerance are recorded among the Asian population, especially women. The study concluded that food allergies are very common now and shellfish is reported as the most common food allergen followed by peanuts, fruits, dairy, and vegetables. The study's major goal was to show that higher rates of awareness and reporting could be a viable explanation for the gender gap. The previous report by Zhou et al. (2013) declared that there has been a significant rise in food allergies in the last decade. In the United States, there were more hospitalized patients with food allergies. According to the European Academy of Allergy and Clinical Immunology (EAACI), due to psychological and biological factors, almost 60% of the allergic patients are women. According to one theory, estrogen makes women more susceptible to diseases that impair the immune system. The occurrence of food intolerance and food allergy was found to be more common in females at 2.9% and 4.2%, respectively, and in Asians, it is 3.6% and 4.3% (Acker et al., 2017). There has been a lack of proof linking maternal diet to childhood allergies, but this is still controversial among health professionals (Gamboni et al., 2013). Researchers are investigating preventive measures as the prevalence of food allergies is increasing day by day. Preventive approaches include the mother's diet throughout pregnancy and breastfeeding, as well as feeding habits such as formula feeding or breastfeeding during infancy (Tasleem et al., 2017). A 2010 study from the Food Allergy Expert panel found that avoidance of maternal allergens during pregnancy was not recommended. A handful of studies directly discussed the effects of peanut consumption during pregnancy. Several limited studies of highly allergic young children have shown a link between peanut ingestion during pregnancy by a mother and peanut allergy. Where the data available are vague, committees of experts have shied away from making recommendations. The scientific community clearly does not know the right response and does not want to offer advice that might turn out to be either incorrect or harmful (Sicherer, 2017).

## 7 FOOD ALLERGY AND FOOD INTOLERANCE

There are significant pathophysiological distinctions between food allergy and food intolerance, resulting in various diagnostic techniques and therapy possibilities, and they are classified based on whether they have an immune component or not (Tuck et al., 2019). Food allergies are the immune response of the body to the protein that is present in food. The body's immune system detects offensive proteins as harmful and enters defensive mode to battle against proteins. Foods considered to be triggering allergic reactions are known as allergens. The main allergens are shellfish, milk, fish, eggs, wheat, peanuts, soy, and tree nuts. They are generally considered “the Big Eight.” Typical symptoms of an allergic reaction include diarrhea, skin irritations, vomiting, nausea, and life‐threatening anaphylaxis which is considered the most severe type of allergic reaction. Many of these symptoms can make you feel ill, but they are not enough to rule out a food allergy. Only an allergy doctor who has been qualified by the board should treat food allergies. Different types of techniques are used by allergists to diagnose a true food allergy. The diagnosis and treatment of food allergies are extensively considered during pregnancy (Acker et al., 2017). It is a chemical reaction that occurs in the body after drinking or eating some food. Food intolerance is more common than food allergy. It is caused by some flavorings, food additives, colorings, and preservatives as well as by various organic chemicals that are naturally present in the food. Some types of food intolerances can also be caused by a lack of digestive enzymes. Lactose intolerance, gluten intolerance, and fructose intolerance are common. Digestive intolerances exist only in the digestive system of the body. Immune system is not involved in them. Food intolerance can occur when our bodies lack specific proteins that are required for the breakdown of certain foods. They can also occur when the digestive system is irritated by a food component. Lactose intolerance is the most widely recognized allergy to food. Food intolerance symptoms include vomiting, headaches, diarrhea, stomach pain, and nausea. Although these symptoms can be similar to those of a food allergy, food intolerances do not result in anaphylaxis. While discomforting, food intolerances rarely become life‐threatening. Diagnosis of food allergy has to be treated by a medical professional, physician, or allergist. There are some other medical tests to confirm food intolerance like a stool acidity test (Acker et al., 2017).

## 8 SKIN ALLERGIES DURING PREGNANCY

Skin allergies are also known as allergic contact dermatitis. It occurs when sensitive or allergic skin comes into contact with an allergen. Immune system cells are responsible for detecting and eliminating foreign bodies such as bacteria and viruses. In most cases, the immune system's response guards against serious diseases. People with skin allergies have an overly sensitive immune system. Proteins contained in medications, latex, food, pollen, and other substances can cause allergic skin rashes and other disorders (Glantz et al., 2004). The offending substances are most irritating to the skin and mucous membranes. Because the substance can travel via the bloodstream to any part of the body, it is realistic to expect a wide range of symptoms. Urticaria, nettle rash, and eczema are some of the most common allergic symptoms on the skin. Eye itching, redness, burning, and swelling can occur on their own or in conjunction with nasal tube irritation. When a person eats specific foods to which he is allergic, he develops canker sores and fever blisters. There are three common categories of skin conditions that are related to pregnancy. Benign skin conditions from normal hormonal changes, preexisting skin conditions, and pregnancy‐specific dermatoses (Tunzi & Gray, 2007). Skin problems can also cause by hormones during pregnancy. Itching, acne, and melasma are the most common skin allergy conditions. Some other familiar problems are redness of palms, stretch marks, edema (fluid retention), varicose veins, and hair loss during pregnancy. Darkening of the skin is also very common among pregnant women. A dark line “linea nigra” develops down the middle of the stomach for some women, which normally fades after pregnancy. Treatment of skin allergies is to avoid the allergen and take preventive medicine such as antihistamines (Kaaja & Greer, 2005).

## 9 CONCLUSION

Proper nutrition is of utmost importance. A well‐rounded and nutritious diet is recommended for pregnant women to avoid the risks of food allergies and to cope with the already existing food allergies. Some researchers recommend that pregnant women try natural remedies at home. The food the mother eats during pregnancy might contribute to her and also the child's immunological profile. Allergies are very prevalent during pregnancy, although not all pregnant women who have them suffer from them for a long time. Many women with no recognized previous allergies only report symptoms during pregnancy. The extent of responses to the allergies depends upon the hypersensitivity to the allergen. Health Care Experts concluded that a balanced diet is of great importance before the pregnancy, during pregnancy, and even after the pregnancy to avoid the risks of having any type of food allergies. To prevent the start of allergic diseases in infants, international guidelines recommended the avoidance of allergenic foods during breastfeeding and pregnancy. Researchers have also found that cesarean delivery might raise the risk of infant allergies. Peanut allergy is the most prevalent and highly noted food allergy. In this report, it is concluded that a pregnant woman should take a balanced diet and avoid consuming known allergenic foods to minimize the risk of complications. There is no conclusive evidence for protecting the child from not having a food allergy if the mother is suffering from some kind of food allergy. However, there is no evidence that avoiding them reduces the likelihood of allergies in children. According to some studies, breastfeeding may assist in preventing food allergies.

## AUTHOR CONTRIBUTIONS

**Tabussam tufail:** Writing – original draft (equal). **Yusra Rasheed:** Writing – review and editing (equal). **Huma Bader Ul Ain:** Conceptualization (equal); data curation (equal). **Muhammad Umair Arshad:** Supervision (equal). **Muzzamal Hussain:** Writing – review and editing (equal). **Nadeem Akhtar:** Software (equal); visualization (equal). **Shamaail A. Saewan:** Resources (equal); supervision (equal).

## FUNDING INFORMATION

No funding was received to undertake the study.

## CONFLICT OF INTEREST STATEMENT

The authors declare no conflict of interest.

## Data Availability

The data that support the findings of this study are available on request from the corresponding author.
